# Configuration and Performance of a Mobile ^129^Xe Polarizer

**DOI:** 10.1007/s00723-012-0425-7

**Published:** 2012-11-10

**Authors:** Sergey E. Korchak, Wolfgang Kilian, Lorenz Mitschang

**Affiliations:** Physikalisch-Technische Bundesanstalt, Abbestr. 2-12, 10587 Berlin, Germany

## Abstract

A stand-alone, self-contained and transportable system for the polarization of ^129^Xe by spin exchange optical pumping with Rb is described. This mobile polarizer may be operated in batch or continuous flow modes with medium amounts of hyperpolarized ^129^Xe for spectroscopic or small animal applications. A key element is an online nuclear magnetic resonance module which facilitates continuous monitoring of polarization generation in the pumping cell as well as the calculation of the absolute ^129^Xe polarization. The performance of the polarizer with respect to the crucial parameters temperature, xenon and nitrogen partial pressures, and the total gas flow is discussed. In batch mode the highest ^129^Xe polarization of P_Xe_ = 40 % was achieved using 0.1 mbar xenon partial pressure. For a xenon flow of 6.5 and 26 mln/min, *P*_Xe_ = 25 % and *P*_Xe_ = 13 % were reached, respectively. The mobile polarizer may be a practical and efficient means to make the applicability of hyperpolarized ^129^Xe more widespread.

## Introduction

The nuclear spin polarization of noble gases becomes transiently enhanced by four to five orders of magnitude when the angular momentum from laser photons is transferred to the nuclear spins by spin exchange processes with optically pumped alkali metal atoms [1]. Nuclear magnetic resonance (NMR) measurements on such hyperpolarized noble gases are boosted in sensitivity, offering a great potential for spectroscopy and imaging of molecules, materials and organisms [2–4]. Their bio-compatibility and the ability and ease to introduce them non-invasively into cell culture, animals, and humans [5, 6] render hyperpolarized noble gases particularly suitable for biomolecular research and clinical applications. Magnetic resonance imaging (MRI) of human lungs has been developed with the focus on ^3^He [7, 8], because of the isotopic pureness and the high gyromagnetic ratio which lead when combined with effective polarization techniques to the highest achievable magnetization among the noble gases. Nevertheless, a wide variety of applications using hyperpolarized ^129^Xe are currently explored due to several reasons. Natural xenon is abundant, inexpensive, and, nowadays, isotope enrichment is affordable. The spin-1/2 nucleus ^129^Xe (natural abundance 26 %) can keep the polarization from tens of seconds in solution [9] and in vivo [10] up to 100 h in the gas phase [11]. The polarizability of its electron shell accounts for a millimolar solubility in aqueous solution with chemical shift sensitivity to the microscopic or molecular environment higher than 200 ppm [12], as well as for a stable binding via dispersion forces to molecular host structures [13]. Accordingly, applications of dissolved hyperpolarized ^129^Xe range from the investigation of protein structure and dynamics in vitro [14] to in vivo studies of blood flow and tissue perfusion in animals [15] as well as in humans [10]. Also, specifically functionalized molecular cages have been designed to house xenon with the prospect to develop highly specific and sensitive NMR probes for bio-analytics or contrast agents for clinical imaging [16].

The basic requirement for all these applications is, of course, the availability of hyperpolarized ^129^Xe. Polarization equipment usually resides in some proximity to the NMR instrumentation, typically a next-door arrangement, to allow for the fast transfer of xenon with small relaxation losses. Of particular advantage is a mobile polarizer, i.e., a self-contained, stand-alone, and transportable device. Such instrumentation can be placed within the laboratory in the optimal location, with regard to either sensitivity or handling. A mobile polarizer can also be taken temporarily to any facility where one is interested in using hyperpolarized ^129^Xe, avoiding the need for costly stationary equipment and not hindering proper operation of the facility at other times. Mobile polarization equipment to be used for human lung imaging has been described recently [17]. The main features are operability in a clinical environment and the production of large volumes of hyperpolarized ^129^Xe over extended periods of time as is required for human application. Here, we present a mobile polarizer for use in research settings. The compact device generates medium amounts of hyperpolarized ^129^Xe for in vitro spectroscopic applications or in vivo experimentation with small animals. Besides the general layout of the instrument, an online NMR module for observing the generation of hyperpolarized ^129^Xe and the overall performance of the polarization process are described.

## Experimental

### The Mobile ^129^Xe Polarizer

**Fig. 1:**
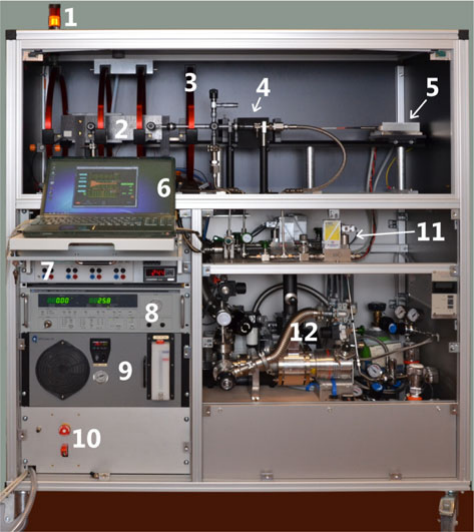
Image of the mobile polarizer. *1* Laser warning light, *2* oven with SEOP cell inside, *3* magnet coil, *4* optics, *5* laser head, *6* laptop, *7* four channel power supply (electronics and electromagnet), *8* laser power supply, *9* laser chiller, *10* emergency laser shut-off, *11* gas flow regulation system, *12* vacuum system (rough pump, turbo pump)

The polarizer is a stand-alone, self-contained device requiring only supplies of pressurized air and two independent 16 A wall sockets at 230 V for the generation of hyperpolarized ^129^Xe. It is designed as a modular system assembled together on an aluminum rack on wheels measuring 130 × 64 × 152 cm (W × D × H) with a total weight of ~300 kg (Fig. 1). The upper part, spacious and conveniently placed for hands-on operation, houses the elements crucial for spin exchange optical pumping (SEOP): the laser with optical elements, the pumping cell, a Zeeman field (*B*_0_) generating coil system, the online NMR module, and the glass tubing for delivery and release of gases. Here, a light-tight shielding (5 mm anodized aluminum) allows for standard operation while for safety reasons operation at open shield is only possible after setting the laser interlock system to a ‘laser-class 4’ mode requiring instructed operators. Any supplies for power, gases and cooling, a vacuum system, the electronics of the online NMR, and a laptop computer for running the system are put together in a most compact manner in the lower part of the rack.

#### Gas Mixing

Four gas cylinders for He, N_2_, natural Xe (26 % ^129^Xe) and enriched Xe (91 % ^129^Xe) are fixed at the base of the rack. After being released from the bottles using cylinder regulators the gases are fed through purifiers to remove traces of moisture and oxygen (Oxisorb^®^, Spectron Gas Control Systems GmbH, Frankfurth, Germany). Afterwards, three digital mass flow controllers (Model F-200CV, Bronkhorst High-Tech BV, Ruurlo, The Netherlands) are used to set the absolute gas flow in a wide range^1^ before two mixing chambers (Bronkhorst High-Tech BV) for uniform blending are passed. Thus, the partial gas pressures of the mixture entering the SEOP cell can be chosen quite freely while an overall constant pressure is maintained by a digital pressure regulator (Model P-702CV, Bronkhorst High-Tech BV) at the very end of the gas stream.

#### Spin Exchange Optical Pumping Cell

**Fig. 2:**
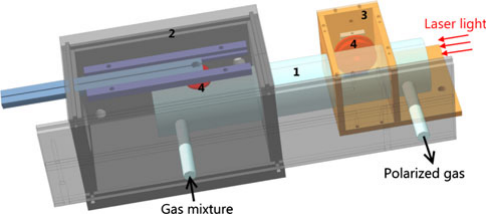
Construction of the SEOP cell (*1*) with movable oven (*2*), brass RF shield (*3*) and NMR transmit–receive coils (*4*)

The SEOP cell (Fig. 2) is a custom-made Duran glass cylinder (Hellma GmbH and Co. KG, Müllheim, Germany). It is designed to withstand pressure differences up to 5 bar and has an inner diameter and inner length of 37.6 and 210 mm, respectively. Inlet and outlet pipes are attached 1 cm away from the optically flat end windows. Some droplets (less than 0.2 g) of rubidium are deposited inside the SEOP cell through the gas inlet pipe using a pipette within a glove box under nitrogen overpressure. The desired rubidium vapor density is obtained by heating up the SEOP cell within a hot-air oven (custom made of PEEK material) which was designed to be moveable along one half of the cell for the optimization of SEOP. The temperature of the cell is stabilized by a PID controller (CN77000 series, Newport Electronics, Inc., Santa Ana, CA, USA) connected to a thermocouple registering the temperature of the SEOP cell surface next to the Rb droplet. The oven is located around the inlet pipe (Fig. 2 lhs) heating only part of the cell as described by [18]. The glass cell is air tight with respect to the oven due to several layers of Teflon tape. The part of the cell outside the oven (Fig. 2 rhs) is cooled by room temperature forced air flow through the radiofrequency (RF) shield of the online NMR. The temperature gradient from the gas inlet to the gas outlet is measured by three additional thermocouples along the SEOP cell. The incoming laser light propagates in reverse direction to the temperature gradient. For such a configuration Rb vapor density is high at the gas inlet and low at the gas outlet which is advantageous compared to an evenly heated SEOP cell [17, 18]. Rubidium is separated by condensation to the walls before the gas leaves the SEOP cell, prohibiting ^129^Xe spin polarization losses by back transfer to unpolarized Rb in regions outside the cell where no laser light is available. Also, the well-known effect of ‘Rb runaway’ [19, 20] leading to an excessive rubidium density and generation of Rb polarization only within a rather small volume where the laser enters the cell is avoided. The laser-light absorption within the SEOP cell is monitored by measuring the transmitted laser-light power (PowerMax-USB PM150-50C, Coherent, Santa Clara, CA, USA) and the optical spectrum (HR2000, Ocean Optics, Dunedin, FL, USA) behind the oven. The window at the rear side of the oven is anti-reflection coated to avoid power losses when light is passing, so that light from a second high-power laser can be applied for two-sided pumping.

#### Magnet

The design of the Zeeman field generating coil system is based on analytical field calculations. Four coils (from two pairs of commercial Helmholtz coils, PHYWE, Göttingen, Germany; 40 cm diameter, 154 windings each coil) with separations of *d*_12_ = 16, *d*_23_ = 11.7, and *d*_34_ = 16 cm, are placed with the symmetry axis parallel to the laser beam. The system is driven by a single power supply, where the total current is fed through the two outer coils but only half of the current through the inner ones by a parallel connection. The *B*_0_ field is sufficiently homogeneous with a calculated relative field gradient smaller than 2 × 10^−4^ cm^−1^ (0.07 mT/m for field strength *B*_0_ = 3.6 mT at maximum current of 5 A) along 28 cm on the central axis.

#### Laser

A high-power laser-diode system with a spectral narrowed linewidth (Brightlock Ultra-500, 75 W at 794.8 nm and linewidth* Δλ* ≈ 0.5 nm, QPC Lasers by Laser Operations LLC, Sylmar, CA, USA) is used whose wavelength can be tuned by changing the temperature of the on-die wavelength selective grating inside the laser head [20]. For the maximum output power our system has a linear dependence of the wavelength λ on the laser-head temperature *T*_Laser_ (in °C) as measured to be λ = 793.66 nm + *T*_Laser_  × 0.073 nm/°C within the range of 14–20 °C. The high linear polarization of the laser light emitted from the diode is partially destroyed in the 30-cm long glass fiber connecting to the collimator. Therefore, only 80 % of the incident laser power is transmitted by the linear polarizing beam-splitter cube on axis. After passing the quarter-wave plate the laser beam is adapted to the diameter of the SEOP cell using two lenses in Kepler configuration. The incident laser power on the SEOP cell is *P*_0_ ≈ 50 W, the transmitted laser power through the cold SEOP cell (i.e., negligible Rb vapor) is reduced to ~30 W due to reflections from the uncoated cell windows and a slightly larger laser-beam diameter compared to the SEOP-cell diameter. We do, however, take the cold-cell measurements as a reference for the determination of the transmission during SEOP. Thus, we can estimate that the laser power entering the SEOP cell is between 35 and 40 W.

### The Online NMR Module

From our experience online monitoring of the generation of ^129^Xe polarization during SEOP is crucial for optimization of the pumping process itself [21]. For this purpose determination of relative polarization levels is sufficient. Knowledge of absolute ^129^Xe polarization (*P*_Xe_) leaving the SEOP cell is, however, required for quantification of losses that occur downstream until the gas is finally applied in an investigation. These purposes are served by an online NMR system, a digital spectrometer equipped with an RF pulse generator, a *Q* factor switch, a signal receiver, and a signal processor operating at low frequencies in the range of 10–200 kHz.

The online NMR is made out of just three hardware components: first, a hand-wound coil for RF transmission and reception; second, a home-made analog circuitry for transmit–receive switching and signal amplification (adapted from [22]); and third, a commercial multi I/O card (NI USB-6341, National Instruments, Austin, TX, USA) for digital control, pulse generation, and data acquisition. The use of only a single I/O card assures phase coherent data acquisition with respect to RF excitation as is required for signal averaging. The operator control of the online NMR is integrated into the LabVIEW operation program of the polarizer and may, thus, be used in a feedback mode to maintain a stable polarization level.

The online NMR can be operated with two different coil setups. Both arrangements comprise a single transmit–receive surface coil which is mounted ≈1 mm off the pumping cell and orthogonally to the main *B*_0_ field-direction (Fig. 2). In contrast to volume coils [18], small surface coils destroy only a minor fraction of the hyperpolarization by RF excitation which may be negligible even in a continuous flow mode and they allow for spatial-dependent measurements.

In one arrangement, the coil is placed outside the oven right before the gas outlet of the SEOP cell to monitor the final ^129^Xe polarization. The flat coil made out of 600 turns of copper wire (0.23 mm diameter) has an outer and inner diameter of 33 and 13 mm, respectively, and performs with a quality factor *Q* = 14 having an inductance of 17.5 mH and a resistance of 40 ohm. A brass shield encasing the coil and partially surrounding the SEOP cell reduces the pick-up of ambient noise and RF intrusion by a factor of 6. The temperature within this shielding and thus of the coil is kept constant at room temperature by forced air flow.

In a second arrangement, another coil is mounted on a slider inside the oven to facilitate localized measurements along the SEOP cell, thus providing spatial information on the SEOP process. This coil of 220 turns of copper wire (0.2 mm diameter) and of 20 and 10 mm outer and inner diameter, respectively, operates at *Q* ≈ 20 and has an inductance of 3.55 mH and a resistance of 15 ohm (at room temperature).

For both arrangements the coil operates during acquisition at relatively low *Q* to avoid quick disappearance and distortion of the signal because of strong radiation damping due to the hyperpolarized ^129^Xe gas [23]. A second advantage of such a low *Q* is the short response time for signal detection at low frequency. At short *T*_2_^*^ of the NMR signal (^129^Xe or protons) on the order of a few tens of ms or less due to the inhomogeneity of the Zeeman field generated by the rather small coils of our mobile polarizer, most of the signal would be missed. For example, at high *Q* = 220 used in [24] the response time is 1.7 ms, whereas in our case of the stationary coil at *Q* = 14 and frequency *f*_0_ = 40 kHz the measured response time is just 110 μs.

**Fig. 3:**
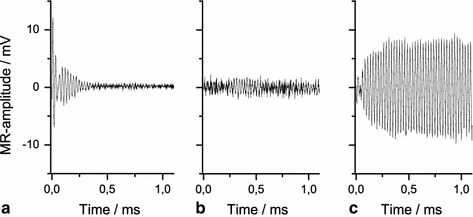
Online NMR signals acquired with the stationary coil outside the oven. Ringing of the coil due to residual transmit current in the average of ten off-resonance acquisitions (**a**), subtraction of the signal shown in **a** from a single on-resonance scan without SEOP (**b**), onset of a typical ^129^Xe FID as subtraction of the signal shown in **a** from a single on-resonance scan during SEOP (**c**)

**Fig. 4:**
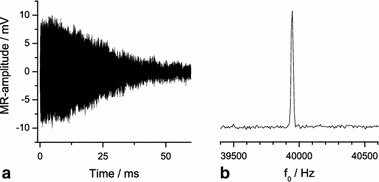
^129^Xe FID acquired with the stationary coil outside the oven (**a**), and the real part of the corresponding spectrum (**b**) at parameter settings *p*_Xe_ = 0.1 bar, *p*_N2_ = 0.2 bar, *p*_He_ = 2.7 bar and total flow rate of 194 mln/min

In transmission mode, however, even such relatively low *Q* is not acceptable for the online NMR. RF radiation will persist some time even after the generating voltage from the multi I/O card has leveled off because of a damped oscillatory exchange of field energy in the resonant circuit. To avoid dominating artifacts, the ring down has to drop well below the NMR signal level which would take several ms at *Q* ~ 14 (at *Q* ~ 10 a ring-down time in the order of 6 ms was found in [25]). For as short a *T*_2_^*^ as ~10 ms, thus, a strong damping of the ring down is crucial. For this purpose the *Q* is actively switched to an even lower value of ~1 by a TTL pulse from the multi I/O card during RF transmission and up to 0.2 ms thereafter. Data acquisition is started after an additional delay of 50 μs. During the low *Q* period a resistance of ~200 ohms in parallel to the coil is enforcing a sufficiently fast release of the stored energy to achieve ring down in ~0.3 ms (Fig. 3a). The residual ring down is now comparable in amplitude to the NMR signal. For even further suppression a difference experiment can be done. First, the signature of the coil ringing alone is acquired by setting *B*_0_ to an off-resonant condition (Fig. 3a). That time series is subtracted from a second on-resonant acquisition (*B*_0_ = 40 kHz × 2π/γ) comprising the ring-down artifact and the desired NMR signal. In the difference time domain signal the ring down is absent (Fig. 3b), and the maximum NMR signal amplitude is established after about 0.4 ms (Fig. 3c). Thus, we were able to detect the ^129^Xe signal with a signal-to-noise ratio of ≈5:1 in the time domain in natural abundance at partial pressures of only 0.1 bar (Fig. 4a).

### Signal Analysis

For control of the ^129^Xe polarization generation we run online NMR acquisitions with the LabVIEW control program displaying the difference time domain signal and its power spectrum together with the original off- and on-resonance transients at the same time. One option to determine amplitude, width and position of the xenon NMR signal (*S*_0_/mV, *T*_2_^*^/ms, *f*_0_/kHz) is the fit of a Lorentzian line shape function to the power spectrum which is strictly valid only for a purely exponentially decaying time domain signal. Due to inhomogeneous broadening that requirement is barely met in our measurements resulting in an overestimation of *S*_0_. As an alternative which takes into account the almost stationary signal amplitude right at the beginning of the time domain NMR signal, a product of an exponential and a Gaussian function (corresponding to a Voigt line shape in frequency domain [26])

1 $$ g(t) = S_{0} {\text{e}}^{{ - \left( {{{(t + \tau_{d} )} \mathord{\left/ {\vphantom {{(t + \tau_{d} )} {T_{2G} }}} \right. \kern-\nulldelimiterspace} {T_{2G} }}} \right)^{2} }} {\text{e}}^{{ - \left( {{{(t + \tau_{d} )} \mathord{\left/ {\vphantom {{(t + \tau_{d} )} {T_{2L} }}} \right. \kern-\nulldelimiterspace} {T_{2L} }}} \right)}} $$

may fit as an envelope function to the magnitude time domain data, calculated from the square root of the sum of the squared real and imaginary components. With our single coil setup we, thus, sample data points at a rate four times the resonance frequency (*f*_s_ = 4*f*_0_) and obtain real and imaginary parts with half that sampling rate by the so-called integer digital conversion [27]. The parameter τ_d_ = 0.25 ms is the delay between the end of an RF pulse and the start of data acquisition. As the time constants *T*_2G_ and *T*_2L_ for the Gaussian and Lorentzian decay, respectively, of similar magnitude (~0.1 s) account for a plateau in the amplitude of the time domain signal over a significant period, the delay τ_d_ can be safely ignored in the fitting (error less than one percent). Both implementations considered here are not sensitive to the phase of the time domain signal and, thus, are more robust than the common approach where the signal amplitude is determined from an in-phase absorption line.

### Proton Calibration for Absolute ^129^Xe Polarization Determination

**Fig. 5:**
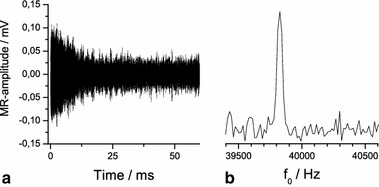
^1^H signal of 6,000 averaged difference experiments (**a**) and real part of spectrum (**b**) for a thermally polarized doped water sample

One important aim of our development of the online NMR is to enable a calibration of the NMR signal in order to estimate the absolute ^129^Xe polarization in situ during SEOP as has been suggested previously [28]. In this way a reference measurement of a well-defined quantity of polarized gas at a high-field NMR instrument with the concomitant difficulty to assign relaxation losses due to refilling and transportation of the gas is avoided, and absolute polarization evaluation could be easily obtained on demand. One common approach, adopted also by us, is to determine a reference signal from a thermally polarized sample using the online NMR. Due to the weakness of our Zeeman field only a high-density sample and fast accumulation are permissible. To this end, the SEOP cell in the polarizer is replaced by an indentical one filled with distilled water. By addition of 6 mM CuSO_4_ (0.1 g CuSO_4_ in 100 ml water) the proton spin lattice relaxation time is reduced to *T*_1_ ~ 0.1 s. A direct comparison of ^1^H and ^129^Xe signals requires the same sensitivity of the respective detection coil in either case. Therefore, the Zeeman field is lowered to *B*_0_ ~ 1 mT to obtain the resonance condition *f*_0_ = 40 kHz also for the water protons, and the very same stationary coil at the outlet of the pumping cell is used for acquisition keeping *Q* at 14 as for the ^129^Xe measurements. To generate an identical excitation profile within the water sample as in the case of ^129^Xe, the excitation pulse of equal amplitude has to be a factor $\gamma_{Xe} /\gamma_{H} \approx 1/3.6$ shorter in duration. The used pulse lengths of 0.5 and 1.75 ms for ^1^H and ^129^Xe, respectively, provide maximal signal in either case as verified experimentally and may still be short enough to neglect relaxation during excitation (*T*_2_^*^ ~ 10 and ~20 ms for ^1^H and ^129^Xe, respectively). The average of 6,000 difference experiments has to be taken to obtain a similar signal-to-noise ratio as in the case of a single difference experiment for hyperpolarized ^129^Xe (see Fig. 5). The total measurement time is 1 h at a repetition time of *TR* = 0.33 s which is sufficient for a complete recovery of the ^1^H thermal polarization.

Under these conditions the ratio of the amplitudes of the ^1^H and ^129^Xe signals is

2 $$ \frac{{S_{{0,{\text{H}}}} }}{{S_{{0,{\text{Xe}}}} }} = \frac{{\gamma_{\text{H}} P_{\text{H}} \rho_{\text{H}} }}{{\gamma_{\text{Xe}} P_{\text{Xe}} \chi_{\text{Xe}} \rho_{\text{Xe}} }}, $$

where for each respective species $\gamma$ is the gyromagnetic ratio, *P* is the polarization, $\rho$ is the molar density (*ρ*_H_ = 110.7 M for ^1^H in water at room temperature), and $\chi_{\text{Xe}}$ is the isotopic abundance of ^129^Xe within the xenon gas (*χ*_Xe_(nat.) = 0.26). The ^1^H polarization at thermal equilibrium in the high temperature approximation valid at room temperature is

3 $$ P_{\text{H}} = \frac{1}{2}\frac{\Updelta E}{kT} = 3.207 \times 10^{ - 9} . $$

Concerning the density of xenon gas, $\rho_{\text{Xe}}$, any uncertainty in the temperature or its variation across the volume affected by RF pulsing will compromise the polarization determination. In our setup detection takes place at the outlet of the pumping cell, well outside the heated oven region. Here, the density of rubidium vapor is small because of the low temperature and consequently laser light is barely absorbed therefore heating of the gas at the location of the coil is avoided. The temperature within the relatively small gas volume affected by the NMR measurement is, thus, homogeneous and can be easily determined as the temperature of the cell walls at the position of the coil. Its constant value, nevertheless, varies between 55 and 75 °C, depending on the setting of the oven temperature. Further assuming xenon to be an ideal gas at low partial pressure *p*_Xe_ and high temperature *T* of our experiments, the molar density becomes $\rho_{\text{Xe}} = {{p_{\text{Xe}} } \mathord{\left/ {\vphantom {{p_{\text{Xe}} } {RT}}} \right. \kern-\nulldelimiterspace} {RT}}$, where *R* is the gas constant. With these assumptions the absolute xenon polarization can be obtained as

4 $$ P_{\text{Xe}} = \frac{{S_{{0,{\text{Xe}}}} }}{{S_{{0,{\text{H}}}} }}\frac{{\gamma_{\text{H}} \rho_{\text{H}} }}{{\gamma_{\text{Xe}} \chi_{\text{Xe}} }}\frac{RT}{{p_{\text{Xe}} }}\; P_{\text{H}} = \frac{{S_{{ 0 , {\text{Xe}}}} }}{{S_{{0,{\text{H}}}} }}\frac{T}{{ \chi_{\text{Xe}}}{p_{\text{Xe}} }}1.07 \times 10^{ - 7} \frac{\text{bar}}{\text{K}}. $$

On practical grounds a proton reference signal $\bar{S}_{0,H} = \left( { 7 4 \pm 2} \right)$ μV is used as derived as the average of *S*_0,H_, determined in the time domain as described in Sect. 2.3, from 13 experiments (Fig. 5).

## Results and Discussion

Our configuration of the mobile polarizer enables optimization of the hyperpolarized ^129^Xe generation with respect to the most important parameters: temperature, pressure, gas mixture, and flow rate. The effects are discussed on the basis of the expression for the ^129^Xe polarization after SEOP of duration *t*_r_

5 $$ P_{\text{Xe}} (t_{r} ) \approx \frac{{\gamma_{\text{SE}} }}{{\gamma_{\text{SE}} + \Upgamma_{\text{Xe}} }}\left\langle {P_{\text{Rb}} } \right\rangle \left( {1 -{e}^{{ - \left( {\gamma_{\text{SE}} + \Upgamma_{\text{Xe}} } \right)t_{\text{r}} }} } \right), $$

where *γ*_SE_ is the rate of spin exchange between ^129^Xe and Rb which is proportional to the Rb number density, Γ_Xe_ is the rate of ^129^Xe polarization loss dominated by wall relaxation, and ‹*P*_Rb_› is the spatial average of Rb polarization [29]. Different gas-flow rates are accounted for by correspondingly different residence times *t*_r_ in the SEOP cell.

### Temperature Dependence

**Fig. 6:**
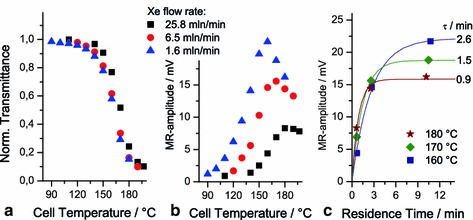
Temperature dependence of laser light transmittance (**a**) and ^129^Xe MR amplitude (**b**) at different xenon flow rates and *p*_Xe_ = 0.1 bar, $p_{{{\text{N}}_{ 2} }} = 0. 2$ bar and *p*_He_ = 2.7 bar. Dependence of MR amplitude on residence time *t*_r_ of ^129^Xe derived from flow rates and SEOP-cell temperature (**c**), solid lines represent the fit of $A\left( {1 - {\text{e}}^{{ - t_{r} /\tau }} } \right)$ in accordance with Eq. (5)

In Fig. 6a, the normalized power of light passing the cell is shown in dependence of the ambient temperature due to the heating of the oven for xenon gas-flow rates of 1.61, 6.46 and 25.84 mln/min.^2^ In the interval from ~150 to ~200 °C a strong intensity drop indicates efficient vaporization of Rb with a concomitant strong increase in light absorption. It is evident that at a given temperature the transmittance increases with flow rate, as comparatively larger portions of Rb are taken with the gas stream, precipitate in the colder part of the cell and are, thus, not available for light absorption. The overall similarity of the transmission curves for different flow rates furnishes evidence of stable conditions concerning particularly temperature or equivalently Rb density prevailing in the experiments.

The ^129^Xe signal amplitudes under the same conditions are presented in Fig. 6b. Each of the temperature dependences first increases strongly with temperature, as *γ*_SE_ rises proportionally to the Rb density (indicated as higher light absorption in Fig. 6a), in agreement with Eq. (5). In addition, at lower gas flow *t*_r_ becomes larger which accounts for a correspondingly higher signal amplitude at a given temperature. From Eq. (5) one would expect *P*_Xe_ to saturate with further increase of temperature, i.e., $P_{\text{Xe}} \approx \left\langle {P_{\text{Rb}} } \right\rangle$, when *γ*_SE_ ≫ Γ_Xe_ and the most efficient spin exchange occur at high Rb density (see Fig. 6c, and discussion below). That scenario, however, is not realistic, as the signal diminishes after a maximum whose position depends on the flow rate (also observed by others [30–32]). With increasing temperature the Rb density becomes ever higher so that above a certain threshold for a given laser power light is completely absorbed in an ever thinner front region of the Rb vapor cloud. That spatially inhomogeneous distribution of polarized Rb becomes steeper with increasing temperature [33] resulting in a decrease in ‹*P*_Rb_› and consequently in a reduction in the overall ^129^Xe signal.

The shift of the maximum ^129^Xe signal toward a higher temperature with increasing gas flow rate (Fig. 6b) can be understood by considering the time dependence of the ^129^Xe polarization build-up as given in Eq. (5) in more detail. In Fig. 6c, the signal amplitude in dependence on the residence time *t*_r_ is shown for three different temperatures (data taken from Fig. 6b). To determine *t*_r_ precisely, we have to account for the expansion and thus for the higher flow of the gas within the SEOP cell due to the higher gas temperature (flow rates of the mass flow controllers are given for normal conditions, i.e., refer to 0 °C). The temperature gradient within the cell is taken coarsely into account by compartmentalization of the cell according to the measurements by four thermocouples, see Sect. 2.1.2. The experimental data are fitted to the function $A\left( {1 - {\text{e}}^{{ - t_{r} /\tau }} } \right)$ derived from Eq. (5) with $A = \frac{{\gamma_{\text{SE}} }}{{\gamma_{\text{SE}} + \Upgamma_{\text{Xe}} }}\left\langle {P_{\text{Rb}} } \right\rangle$ and 1/*τ* = *γ*_SE_ + Γ_Xe_. Both *A* and 1/*τ* depend on Rb density, or, equivalently, on temperature. For all three nominal cell temperatures considered in Fig. 6c (160, 170 and 180 °C) an exponential rise toward a saturation value for a long residence time *t*_r_ is indicated by the data. The higher the temperature the smaller is the saturation value but the rate becomes faster. The curves, thus, cross in between short and long residence times causing a reversal in the temperature dependence of the signal amplitude: the MR signal drops when increasing the temperature from 160 to 180 °C for the longest residence times (i.e., lowest flow rate, blue triangles in Fig. 6b) but rises for shortest residence times (fastest flow rate, black squares in Fig. 6b), accounting for the shift in the signal maxima in Fig. 6b.

Our configuration of an only partially heated cell warrants a particularly stable temperature control of the SEOP. One can change between adjacent temperature settings in Fig. 6b (data not shown) and reproduce the signal amplitudes. In contrast to an all heated SEOP-cell setup (as used in a second, stationary polarizer), only a minor hysteresis effect is observed and a comparatively modest decrease of signal amplitude occurs toward high temperatures.

### Spatial Distribution

**Fig. 7:**
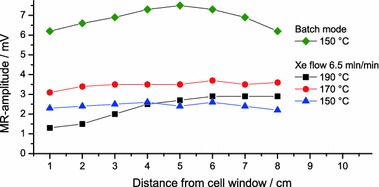
^129^Xe polarization along the heated part of the SEOP cell in dependence of distance from end window of the cell next to the gas inlet. SEOP is done with *p*_Xe_ = 0.1 bar, $p_{{{\text{N}}_{ 2} }} = 0. 2$ bar and *p*_He_ = 2.7 bar at different temperatures and xenon flow rates

The spatial distribution of hyperpolarized ^129^Xe within the pumping cell is presented in Fig. 7. Each data point corresponds to the magnetization determined by the sliding coil positioned at the indicated distance to the end window of the SEOP cell next to the gas inlet. The part encapsulated by the oven is covered completely by the measurements. For a given temperature, the *T*_2_^*^ relaxation times of ^129^Xe along the cell within the oven do not vary by more than 20 % (20 ms < *T*_2_^*^ < 45 ms applies for the whole range of oven temperatures indicated in Fig. 7).

For the constant xenon flow rate of 6.46 mln/min the xenon polarization is rather uniform over the entire range at 150 °C. The situation persists at 170 °C, albeit at a higher signal because of an increase in Rb number density. One would expect a lower polarization level at the gas inlet in comparison to the level further down the cell because in 1 min xenon traverses the heated part of the cell while the rise of its magnetization due to SEOP to a steady state requires ~2 min (see Fig. 6c, also determined independently by Zeeman field reversal and magnetization recovery). The experimentally observed uniformity is probably due to gas convection and turbulent flow which causes homogeneous mixing of already polarized and freshly delivered xenon gas [34]. However, at an even higher temperature the spatially inhomogeneous distribution of Rb polarization as mentioned above comes into play. At 190 °C the polarization build-up is accelerated to less than a minute (Fig. 6c), while local differences cannot be averaged out totally by convection. Thus, the pronounced ^129^Xe polarization gradient reflects at least in part the Rb polarization gradient present at high temperatures (as discussed in Sect. 3.1 for Fig. 6a). It should be mentioned that the gas mixture along the cell due to gas convection nevertheless reduces the SEOP efficiency at high temperatures because highly polarized Xe from the gas exit region transfers its polarization back to less polarized Rb at the gas entrance side where less laser light is available. For comparison, the xenon polarization is shown also in batch mode and at 150 °C. The weak maximum almost in the center of the heated region is presumably due to transverse convection rolls as disclosed by simulations [31].

### Xenon Partial Pressure Dependence

**Fig. 8:**
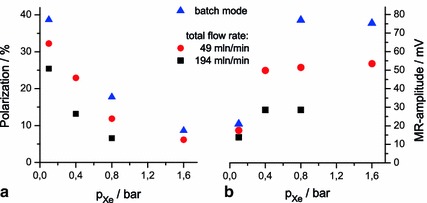
Absolute ^129^Xe polarization (**a**) and MR amplitude (**b**) dependence on composition of the gas mixture and different gas flow rate. For all gas mixtures the nitrogen partial pressure is $p_{{{\text{N}}_{ 2} }} = 0. 2$ bar, and the total pressure is kept at 3 bar

In Fig. 8 the absolute ^129^Xe polarization and the magnetization at optimum temperature are shown in dependence on the fractional xenon content in the gas mixture (varied xenon partial pressure at fixed overall pressure) for batch mode and two different total gas-flow rates. The absolute polarization is determined according to Eq. (4) using the temperature values as measured in the cooled RF shield of the stationary coil used in the experiments (see Sect. 2.4). The ^129^Xe polarization decreases with higher xenon concentration (Fig. 8a) due to the high Rb spin-destruction rate by collisions with xenon. The totally achievable MR signal strength, however, increases with xenon concentration (Fig. 8b) at low xenon pressure because of the increasing number of nuclei contributing to the signal which overcompensates the loss in polarization. That trend, however, levels off as ultimately for a given number of absorbed laser photons the achievable xenon magnetization is limited. Generally, higher yields in polarization as well as magnetization are obtained with reduced flow rate due to the extended residence time in the cell. It is, thus, possible to tailor the signal strength versus the polarization level for particular applications.

### Nitrogen Partial Pressure Dependence

**Fig. 9:**
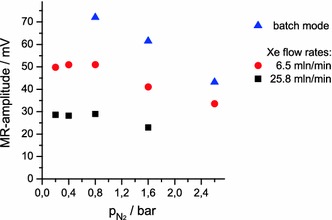
^129^Xe MR amplitude in its dependence on the composition of the gas mixture at three different gas flow conditions. Total pressure is kept at 3 bar, xenon partial pressure is *p*_Xe_ = 0.4 bar

There is little information available about the optimum nitrogen partial pressure in SEOP. Commonly, $p_{{{\text{N}}_{ 2} }} \sim 0. 2$ bar is used [18, 29], but systems with up to $p_{{{\text{N}}_{ 2} }} = 2. 5$ bar are also reported [20]. In our measurements (Fig. 9) we clearly see a decrease in the ^129^Xe MR signals when adding more than one bar nitrogen to the gas at constant overall pressure of 3 bar $\left( {p_{\text{He}} = 3 \,{\text{bar}} - p_{\text{Xe}} - p_{{{\text{N}}_{ 2} }} } \right)$. We presume the effect originates either in the direct interaction of nitrogen with xenon enhancing ^129^Xe relaxation or affecting the efficiency of polarization transfer via spin exchange.

### Total Gas Pressure Dependence

The effect of varying total gas pressure on SEOP for two different xenon concentrations is indicated in Table 1. We used a total gas pressure of 3 bar on the setup assuming sufficient pressure broadening of the Rb absorption line for the narrow bandwidth of the laser light. As can be seen by the laser transmittance there is still light passing the SEOP cell even for very high cell temperatures. This indicates that not all of the light can be used for the SEOP. By increasing the total pressure to 5 bar, nevertheless, hardly any gain for the low xenon partial pressure is observed. For the high xenon concentration at *p*_Xe_ = 1.6 bar a significant improvement occurs, an indication that at high *p*_Xe_ the ^129^Xe polarization is limited by the available laser power due to the strong Rb spin destruction by xenon.

**Table 1:** 3 bar and 5 bar total gas pressure (ptot)

	*p*_Xe_ = 0.1 bar		*p*_Xe_ = 1.6 bar	
	*Φ*_Xe_ = 1.61 mln/min	Batch mode	*Φ*_Xe_ = 1.61 mln/min	Batch mode
ptot = 3 bar	17.5	21.0	53.5	75.3
ptot = 5 bar	18.4	22.8	62.9	87.0

## Conclusions

We developed a stand-alone ^129^Xe polarizer housed in a very compact and mobile rack. Only electrical power and pressurized air supply are needed at the site of operation. The modular arrangement allows for adapting to various applications, i.e., batch-mode production for very small quantities, flow mode for continuous supply or accumulation by freezing for high volume. The mobile polarizer is easy to handle and facilitates the application of hyperpolarized ^129^Xe in a wide range of different and potentially constricted settings.

Another advantage in our setup is the online NMR system for the determination of relative and absolute ^129^Xe polarization during SEOP enabling monitoring of the hyperpolarizing process for control and optimization of parameter settings. Only a commercial multi I/O card and an easy-to-replicate circuitry applicable to a wide range of frequencies are needed to drive different transmit–receive coils. With the shielded coil we were able to determine proton signals for calibration, while using the slidable coil we gained insight into the spatial distribution of SEOP.

The overall performance of the mobile ^129^Xe polarizer is very good, especially as it is not yet fully optimized. So far ^129^Xe polarizations of up to ~40 % in batch mode were determined. For a modest xenon flow of *Φ*_Xe_ = 6.5 mln/min, we reach ^129^Xe polarization of *P*_Xe_ = 25 % using a xenon partial pressure of *p*_Xe_ = 0.1 bar whereas at a four times higher flow of *Φ*_Xe_ = 26 mln/min, we reach *P*_Xe_ = 13 % when using *p*_Xe_ = 0.4 bar. Although yielding half the polarization, the latter setting provides a volume–polarization product that is twice as high in comparison to the first one. It is noteworthy that all the polarizations given are a lower limit (see Eq. 4) as the only uncertainty remaining is the gas temperature which may be elevated by laser heating [35]. This can be cross checked by calibration via thermally polarized ^129^Xe at a high-field spectrometer. A clear result from the presented measurements is that more power of laser light would boost the polarization especially for high xenon partial pressures. To this end we will replace the existing optical fiber with a shorter one in order to reduce the light depolarization and concomitant power losses in the present configuration. Another option to improve our setup is to use a second laser, thus, applying two-sided pumping.

## Footnotes

1 Via software the controller’s full scale flow rate can be adjusted between 40 and 150 % of the nominal value keeping the uncertainties small even for small gas flow.

2 1 mln/min (1 normal ml per min) is the gas flow of 1 ml in 1 min at normal conditions (*T*_n_ = 0 °C and *p*_n_ = 1 atm).
